# The relationship between cannabis use and measures of anxiety and depression in a sample of college campus cannabis users and non-users post state legalization in Colorado

**DOI:** 10.7717/peerj.2782

**Published:** 2016-12-08

**Authors:** Lucy J. Troup, Jeremy A. Andrzejewski, Jacob T. Braunwalder, Robert D. Torrence

**Affiliations:** Department of Psychology, Colorado State University, Fort Collins, CO, United States

**Keywords:** Cannabis, Anxiety, Depression, Recreational cannabis use

## Abstract

As part of an ongoing research program into the relationship between cannabis use and emotion processing, participants were assessed on their level of cannabis exposure using the Recreational Cannabis Use Examination, a measure developed specifically to assess cannabis use in Colorado post state legalization. Three groups were created based on self-reported use: a control group who have never used, a casual user group and a chronic user group. Each participant also completed two measures of mood assessment, the Center for Epidemiologic Studies Depression Scale and the State-Trait Anxiety Inventory. Relationships between cannabis use groups and scores on these measures were then analyzed using both correlations and multivariate analysis of variance. Results indicate a relationship between casual cannabis use and scoring highly for depressive symptomatology on the Center for Epidemiologic Studies Depression Scale. There were no significant relationships between cannabis use and scores on the State-Trait Anxiety Inventory.

## Introduction

The relationship between cannabis use and symptomatology of mood and anxiety disorders is complex. Although a great deal of research exists and continues to grow, the evidence remains contradictory. A large cohort, three-year follow up study suggested that there was a significant relationship between cannabis use and a diagnosis of depression and bipolar disorder, but it was not significantly related to anxiety (Van Laar et al., 2007). In a large cohort longitudinal study, Danielsson and colleagues (2016) showed that when controlling for potential confounding variables such as other drug use, environmental, and social factors, there was no statistically significant relationship between depression and subsequent cannabis use or cannabis use and subsequent onset of depression. In another recent large cohort Australian study, a significant link between cannabis use and depressive symptoms was reported; when controlling for confounding variables the relationship was reduced (Horwood et al., 2012). Another systematic review and meta-analysis of substance abuse disorder and comorbidity with mood disorders showed a significant relationship, with depression being the most significantly related followed by anxiety (Lai et al., 2015). Degenhardt and colleagues (2003) reported a significant increase in negative mood symptoms with heavy cannabis use but not with in frequent cannabis use. In a more recent study attempting to control for the cumulative effects of cannabis use over time, Degenhardt and colleagues (2003) concluded that early onset cannabis use in adolescence extending into adulthood did not increase the likelihood of developing depression, but did increase the risk of anxiety. More recently in a large meta-analysis Kedzior & Laeber (2014) showed a small but significant relationship between cannabis use and anxiety in 10 countries across a large sample of non-institutionalized users. However, it is very difficult to draw conclusions from these large-scale studies based on vast differences in cannabis use patterns and cannabis culture.

Inconsistencies in the literature is better understood when taking into consideration how cannabis use is reported. Phytocannabinoid type and strength is not consistent between studies, and there have been significant changes in the strength of these products post legalization. Phytocannabinoids are being produced that have much higher levels of THC (ElSohly et al., 2016). Products and routes of administration are changing, leading to exposure to much more concentrated forms of THC, for example waxes, oils, and shatters (Lofin & Earleywine, 2014). The phytocannabinoids in these products are poorly controlled and even when tested, measures pertaining to (for example) THC content relative to other phytocannabinoids and terpines, is contradictory and unpredictable and not widely reported in the literature. However, very little research focuses specifically on cannabis use patterns in states were prohibition has been lifted. Most of the studies conducted, then, focus on populations were cannabis use is prohibited or report data with synthetic cannabinoids. In an attempt to clarify some of these confounding variables and inconclusive results from large cohort studies Lev-Ran and colleagues (2014) conducted a systematic review and meta-analysis concluding that cannabis use, particularly chronic use is linked to depression, highlighting the need for more research. For example, positive application of cannabinoids to treat depression focusses heavily on cannabidiol (CBD) which is present in phytocannabinoids in varying degrees of concentration, dependent on specific strains.

The relationship between cannabis and mood disorders is of particular interest to a wide audience including educators and researchers alike. A systematic review of published research between the years 1990–2010, addressing the prevalence of depression in college undergraduates, showed a range of 10–85% with a weighted mean of 30.6%, higher than the general population (Ibrahim et al., 2013). It is as yet unclear the effects legalization will have on cannabis consumption rates in those states that allow medical and recreational use. Hall & Weier (2015) suggested that it would be at least 10 years before the effects are known. This, coupled with the uncertainty of how the multitude of phytocannabinoids present in the cannabis plant affects emotion processing, emphasizes the need for a greater understanding of cannabis use patterns and their relationship to mood and anxiety disorder symptomatology.

Use patterns in adolescents and young adults have become an increasing focus in states where cannabis use is legal. Keith and colleagues (2015) investigated cannabis use patterns in an undergraduate population in the North East United States concluding the prevalence rate in their population ran at 1 in 12 or 8.4%. Recent data from the High Intensity Rocky Mountain Drug Enforcement Agency’s annual report states that Colorado currently has a 74% higher than national average adolescent cannabis use rate (Rocky Mountain High Intensity Drug Trafficking Area, 2016). In July of this year, the Colorado Department of Public Health and Environment launched a large scale survey in Colorado to collect data on the cannabis using populations preferred method of use patterns, with the aim to gather 1,500 responses by Oct 31st 2016 (Colorado Department of Public Health & Environment, 2016). In April 2015, The Denver Post reported that within the government approved testing laboratories there was a significant amount discrepancy in THC levels reported in cannabis in both the Colorado retail and medical market, a discrepancy that has arisen from testing only being required for recreational products prior to 2016. However, as of June this year, the passing of House Bill HB 15-1283 required testing for both Medical and recreational cannabis products. This then creates a population of cannabis users that have a unique profile to cannabis users could differ from the existing published data investigating effect of cannabis on mood.

The mechanisms by which cannabis could be both positively and negatively effecting mood are clearly complex which has implications in light of legalization in four US states, with 24 US states and Washington D.C. permitting the medical use of cannabis. However, mood and anxiety disorders (with the exception of PTSD in 11 states) are currently not an approved condition in any of these states, although Canada has mood and anxiety disorders as approved medical conditions in their Medical Marijuana Access Division (https://medicalmarijuana.ca/for-patients/who-is-eligible). Recent research in our lab (Troup et al., 2016) investigated the residual effects on cannabis and emotion processing in an event-related potential paradigm. We looked at explicit, implicit and empathic response to negative and positive emotional expressions and the effect this had on the P300 Event Related Potential (ERP). The P300 ERP being linked to attention to emotion. Cannabis use patterns were determined using the Recreational-Cannabis Use Evaluation (R-CUE), a questionnaire developed specifically for assessing cannabis use in post-legalization Colorado. Cannabis use led to a reduced P300 in cannabis users in implicit and empathic emotional processing for negative emotional expressions (Troup et al., 2016).

We therefore conducted a follow up study with a different population to see if there is a relationship in our undergraduate college population between cannabis use and mood. Our study places a heavy emphasis on external validity, where reported residual cannabis use reflects the environment that is occurring in and therefore aims to represent an ecologically valid snap shot of cannabis use in our student population. This population has been shown according to various agencies, discussed above, has a higher use rate than other states for adolescents and young adults. We used the R-CUE to compare cannabis users to non-users responses on the The Center for Epidemiological Studies depression scale (CES-D) and the Stait Trait Anxiety Inventory (STAI). The CES-D is considered both reliable and valid measure of depressive symptomology. Scores above 16 on the 20 item scaler are considered to indicate an individual being at risk of a diagnosis of clinical depression (Radloff, 1977). The test is considered to be both sensitive and reliable, with good internal validity (e.g., Lewinsohn et al., 1997). The CES-D is a 20 item questionnaire that can be interpreted in four factors, In a meta-analysis, Shafer (2006) concluded that there was little variability across the factor analysis of the studies reviewed, and the findings of the meta-analysis were consistent with Radloff’s initial factor analysis (Radloff, 1977; Shafer, 2006). However, Carleton et al. (2013), in a more recent review including both undergraduate, community and clinical populations, question the 20-item four factor model and, although its reliability was upheld, suggest a possible modification to a 3-factor 14-item revision (Carleton et al., 2013). The STAI is a measure that assesses anxiety both in terms of situation anxiety (state) or as a person variable (trait; Spielberger et al., 1983). It is considered a reliable measure of anxiety and widely used (e.g., Barnes, Harp & Jung, 2002). There has been some criticism of its ability with the trait portion to distinguish between anxiety and depression (e.g., Bados, Gómez-Benito & Balaguer, 2010; Beiling, Antony & Swinson, 1998). Despite these criticisms it is still considered a reliable and valid measure (Julian, 2011; Spielberger, 2010), with the state portion of the measure having obviously lower reliability that the trait portion (Julian, 2011).

It is also important to note that it we are looking at the residual effects of cannabis use, and administration of specific doses of isolated phytocannabinoids was not a goal of this study, nor was the administration of any specific dose of recreational or medical cannabis. It should also be noted that this study serves as a population pilot for future research using ecological approaches to the possible contributions cannabis use has to pre-mood/anxiety disorder symptoms within the population; therefore, our sample size will be smaller than typical ecological research. Based on our previous research (Troup et al., 2016) we expected to see a significant relationship between cannabis use and scores on mood and anxiety measures, with individuals who are heavy cannabis users showing a significantly higher incidence of anxiety and depression scores consistent with the cut offs described by the STAI and CES-D in the literature. Further, exploratory analysis will allow us to look more closely at these relationships as drivers for future research directions.

## Materials & Methods

### Participants

One hundred and seventy-eight participants were recruited from the undergraduate population at Colorado State University, and received Psychology course credit for participation. All participants underwent screening (via self-report) for neurological disorders and significant brain injury, mental health, both personal and family history, mood and mood related disorders and substance abuse disorders. Current prescription medications, caffeine intake, and tobacco, cannabis, alcohol, and other drug use were screened for in two time intervals (eight and 24 h) by self-report. There was minimal exposure to other substances with only 11.5% using tobacco in the past 8 h (*n* = 19), 4.2% using cannabis in the past 8 h (*n* = 7), and only one participant had used alcohol in the past 8 h. Caffeine was the substance that had the most reported use with 46.3% of participants using it in the past 8 h (*n* = 76). The study was approved by the Colorado State University Office of Research Integrity and Compliance, Institutional Review Board (IRB) protocol ID number 12-3716H.

### Procedure

Participants were asked to give written consent and complete a general demographic questionnaire. They were then asked to complete the following questionnaires designed to screen for mood and emotion processing disorder symptomatology: the CES-D, to screen for pre-depressive symptoms (Radloff, 1977) and the State portion of the STAI, to screen for state-related pre-anxiety symptoms (Spielberger et al., 1983). The participants were also asked to complete the Interpersonal Reactivity Index (IRI), to screens for self-reported dispositional empathy in four separate but related dimensions: perspective-taking, fantasy, empathic concern, and personal distress (Davis, 1983) and the Positive and Negative Affect Scale (PANAS), to measure current affective state, which is consistent with previous cannabis research (Gruber, Rogowska & Yurgelun-Todd, 2009). However, to reduce complexity in the data our analysis focused on the CES-D and STAI. Finally, they completed the R-CUE, a self-reported cannabis use questionnaire developed to address cannabis use in Colorado post legalization (Troup et al., 2016). The R-CUE is a measure of cannabis use patterns developed specifically for research in our laboratory post cannabis legalization in Colorado. It is a detailed descriptive tool that evaluates usage and very specific questions about how cannabis is being used (see Appendix [i] for a copy of R-CUE). After completing the study participants were debriefed. The groupings based on these measures to be included in analysis can be found (see Table 1).

**Table 1:** Cannabis use and mood groupings according to CES-D, STAI and R-CUE.

**Group**	**N**
Depressed users	51
Anxious users	54
Non-depressed users	39
Non-anxious users	36
Depressed non-users	30
Anxious non-users	32
Non-anxious non-users	34
Non-depressed non-users	36

### Analysis

We performed two separate analyses. Firstly, to better investigate the relationship between cannabis use, depression and anxiety, a 2 (CES-D and STAI) × 3 (non-users, casual users, and chronic users) Multivariate Analysis of Variance (MANOVA) was conducted. This was followed by an exploratory correlational analysis. Participant data was excluded if any portion of the questionnaire series was missing (*n* = 30, 16.8% of collected data). The remaining 148 data sets were separated into groups based on their respective user classifications and CES-D /STAI scores. For the CES-D 16 or greater considered the participant to show pre-depressive symptoms (pre-depressed) and for the STAI 39 considered the participant to exhibit pre-anxiety symptoms (pre-anxious),” consistent with norms associated with these measures (Julian, 2011). While there were other measures included in our questionnaire packet, the hypotheses of this study focus on the CES-D and STAI survey scores. Scores on the R-CUE were used to determine cannabis use and user profiles and groupings (casual and chronic) were consistent with previous research (e.g., Degenhardt, Hall & Lynskey, 2003; Morgan et al., 2012; Troup et al., 2016); a participant was considered a chronic user if they used once a week or more, less use was considered casual. Eight groups in total were created based on frequency for creating categories of cannabis use, and cutoffs for categorizing anxiety and depression: pre-depressed users, non-depressed users, pre-depressed non-users, non-depressed non-users, pre-anxious users, non-anxious users, pre-anxious non-users, and non-anxious non-users. Each participant was included in two groups, total; one based on their STAI score and the other based on their CES-D score.

## Results

**Figure 1:**
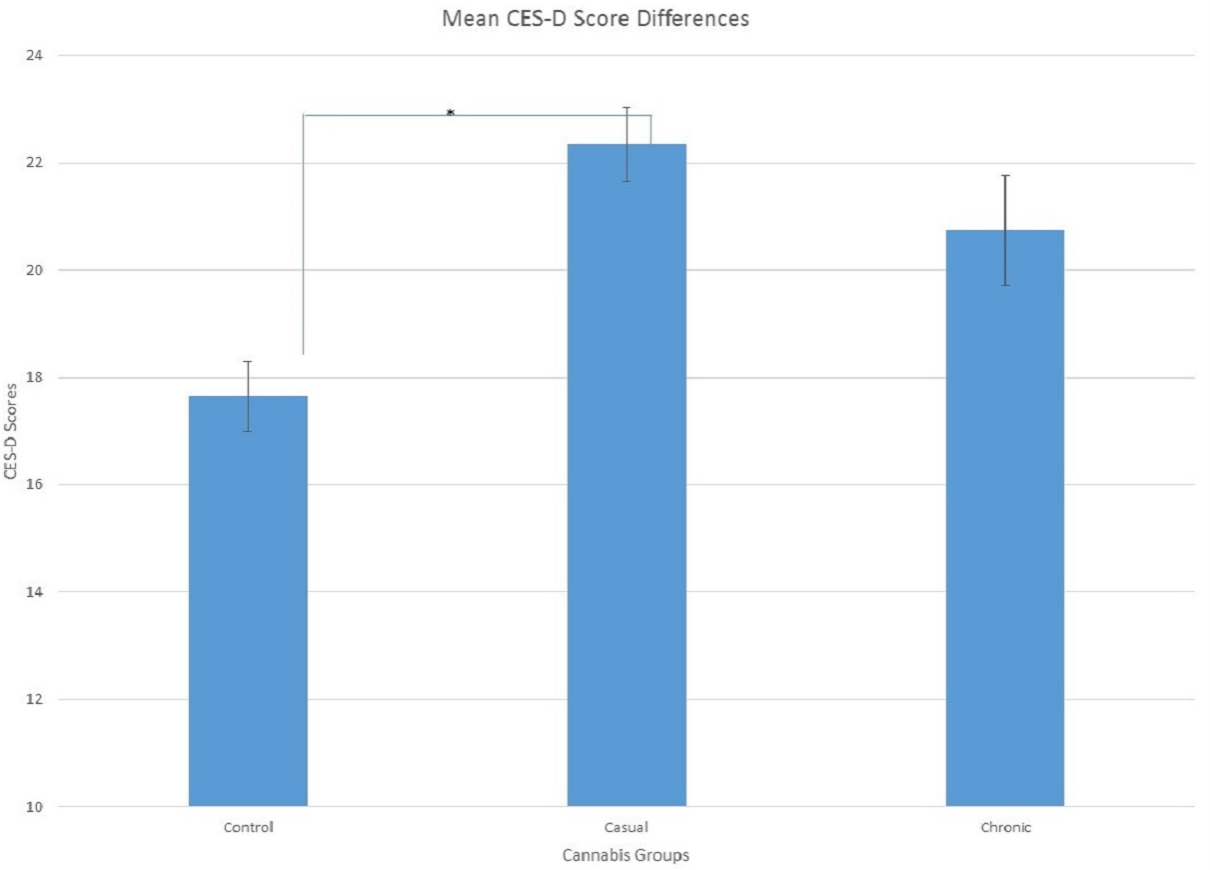
Mean Scores on the CES-D showing significant differences between non-cannabis users and casual cannabis users, *p* < 0.05 as indicated by the (*).

**Figure 2:**
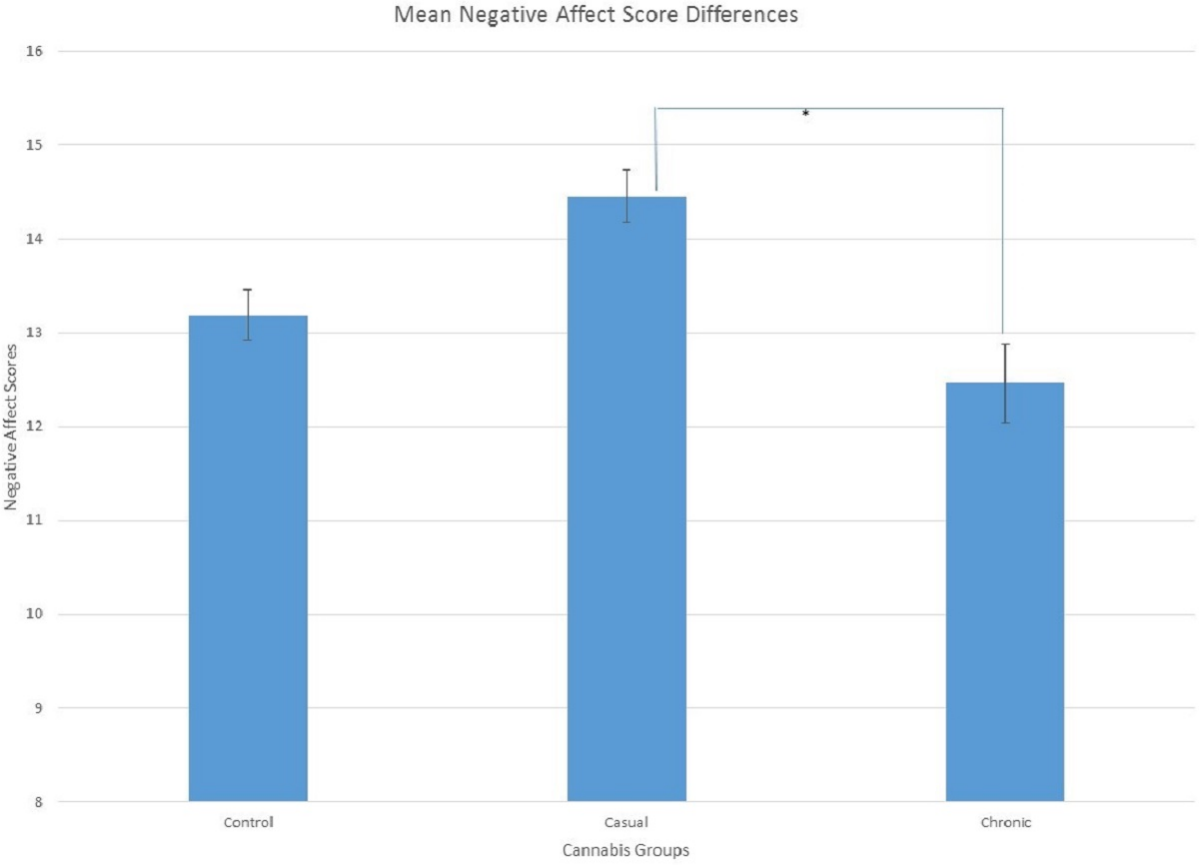
Mean Scores showing significant differences between casual and chronic cannabis users for negative affect, *p* < 0.05 as indicated by the (*).

Data was analyzed using MANOVA was used to examine group differences on the questionnaires. There was a significant difference in the CES-D scores between the cannabis groups, *F*(2, 154) = 3.09, *p* = .015, ${\eta }_{p}^{2}=.039$, sphericity was assumed. Least significant difference (LSD) determined that the non-cannabis users (*M* = 17.65, *SE* = 1.32) had lower scores on the CES-D than the casual cannabis users (*M* = 22.34, *SE*= 1.39; *p* < .05). Chronic cannabis users (*M* = 20.75, *SE* = 2.05) were not different than either group. Differences in negative affect scores between cannabis groups were approaching significance, *F*(2, 154) = 2.37, *p* = .097, ${\eta }_{p}^{2}=.03$. Since the effect size was small to medium, the results from LSD test was then examined. Casual cannabis users (*M* = 14.46, *SE* = 0.56) had significantly greater scores on negative affect than the chronic cannabis users (*M* = 12.46, *SE* = 0.83; *p* < .05; see Figs. 1 and 2). Non-cannabis users (*M* = 13.19, *SE* = 0.53) were not different in either group.

Following from this a second exploratory analysis investigated the relationships between cannabis use groups and scores on the CESD and STAI. The R statistical analysis program (R Core Team, 2013) was used to create matrices of linear correlations for each of the variables within each group and a series of correlograms. In the depressed user group, an increase in user frequency lead to a decrease in STAI scores *r*(49) = −.31, *p* = .027 In the pre-anxious user group, an increase in user frequency was correlated with a decrease in CES-D scores, *r*(52) = −.30, *p* = .029. In users that didn’t qualify for our pre-depressed or pre-anxious groups, an increase in frequency of cannabis use was significantly correlated with a decrease in positive affect. This effect was seen in both non-depressed users, *r*(37) = −.40, *p* = .012 (see Table 2) and non-anxious users, *r*(34) = -.34, *p* = .042.

**Table 2:** Showing significant (*p* <0.05) correlations (in dark red) for depressed and anxious non-users and the lighter color showing a trend towards significance for the CES-D and STAI, with D, Depressed; A, Anxious.

Depressed/Anxious non users				
	CES-D	STAI	PosAffect	NegAffect
Years since				
	CES-D	D/A		D/A
		STAI		D
			PosAffect	
				NegAffect

**Notes.**

Dark red indicates significant correlation at 0.05 alpha value.

Light red indicates trending towards significance.

Many of the groups had common correlations in negative mood and personality measures. First, CES-D scores were positively correlated with STAI scores in six of our eight groups: Pre-depressed users, *r*(49) = .31, *p* = .027; pre-depressed non-users, *r*(28) = .47, *p* = .003; pre-anxious users, *r(52)* = .32, *p* = .018; pre-anxious non-users, *r*(30) = .37, *p* = .034; non-anxious users, *r*(34) = .50, *p* = .001; and non-anxious non-users, *r*(32) = .62, *p* < .001. Additionally, CES-D scores were also positively correlated with negative affect scores in six of our eight groups: pre-depressed users, *r*(49) = .57, *p* <.001 ;pre-depressed non-users, *r*(28) = .39, *p* = .017; non-depressed users, *r*(37) = .38, *p* = .017; non-depressed non-users, *r*(34) = .34, *p* = .042; pre-anxious users, *r*(52) = .74, *p* < .001; and pre-anxious non-users, *r*(30) = .41, *p* = .020. (see Tables 3 and 4)

**Table 3:** Showing significant (*p* < 0.05) correlations (in dark red) for depressed and anxious users and the lighter color showing a trend towards significance for the CES-D and STAI, with D, Depressed; A, Anxious.

Depressed/Anxious users						
	Use frequency	Monthly use	CES-D	STAI	PosAffect	NegAffect
Years since	D/A	D/A				
	Use frequency	D/A	A	D		d/a
		Monthly use				
			CES-D	D/A	d/A	D/A
				STAI		D/A
					PosAffect	NegAffect

**Notes.**

Dark red indicates significant correlation at 0.05 alpha value.

Light red indicates trending towards significance.

**Table 4:** Showing significant (*p* < 0.05) correlations (in dark red) for non-depressed and anxious users and the lighter color showing a trend towards significance for the CES-D and STAI, with D, Depressed; A, Anxious.

Non depressed/Anxious users						
	Use frequency	Monthly use	CES-D	STAI	PosAffect	NegAffect
Years since	a	a				
	Use frequency	D/A	d		D/A	
		Monthly use				A
			CES-D	A	a	D
				STAI	a	d
					PosAffect	NegAffect

**Notes.**

Dark red indicates significant correlation at 0.05 alpha value.

Light red indicates trending towards significance.

**Table 5:** Showing significant (*p* <0.05) correlations (in dark red) for non-depressed and anxious non-users and the lighter color showing a trend towards significance for the CES-D and STAI, with D, Depressed; A, Anxious.

Non depressed/Anxious non-users				
	CES-D	STAI	PosAffect	NegAffect
Years since		a	D	
	CES-D	A		D
		STAI		D/A
			PosAffect	
				NegAffect

**Notes.**

Dark red indicates significant correlation at 0.05 alpha value.

Light red indicates trending towards significance.

Finally, STAI scores were positively correlated with negative affect scores in five of our eight groups: pre-depressed users, *r*(49) = .56, *p* < .001; pre-depressed non-users, *r*(28) = .46, *p* = .010; non-depressed non-users, *r*(34) = .47, *p* = .003; pre-anxious users, *r*(52) = .53, *p* < .001; and non-anxious non-users, *r*(32) = .39, *p* = .022. When analyzing our empathy subscales, empathic concern was positively correlated with perspective-taking in seven of our eight groups: pre-depressed users, *r*(49) = .41, *p* = .002; pre-depressed non-users, *r*(28) = .48, *p* = .007; non-depressed users, *r*(37) = .39, *p* = .014; non-depressed non-users, *r*(34) = .43, *p* = .008; pre-anxious users, *r*(52) = .51, *p* < .001; pre-anxious non-users, *r*(30) = .67, *p* < .001; and non-anxious non-users, *r*(32) = .36, *p* = .036. (see Tables 2 and 5).

Interestingly, empathic concern scores were positively correlated with personal distress scores in both non-depressed non-users, *r*(34) = .42, *p* = .011 and non-anxious non-users, *r*(32) = .39, *p* = .022.

## Discussion

The results suggested that cannabis use had an effect on measurements of mood disorder symptomatology. In particular, those who used cannabis less frequently, the casual user group, had the strongest correlations with overall score and negative affect on the CES-D. Interestingly there was no significant relationship to pre-anxiety symptoms in the cannabis user groups when compared to controls.

As stated in the introduction, the relationship between mood and cannabis use is both contradictory and difficult to assess. Our data and our exploratory analysis shows an interesting relationship between infrequent cannabis use and potential relationships with mood. This supports the literature suggesting that cannabis use has a relationship with scoring highly for pre-depression (Danielsson et al., 2016; Horwood et al., 2012; Van Laar et al., 2007), as defined by scores on the CESD. However, our data reflect a different relationship between infrequent and frequent use and mood. The literature supporting negative mood outcomes from cannabis use report a “dose dependent” relationship with higher levels of cannabis use leading to the greatest deficit in mood (Degenhardt, Hall & Lynskey, 2003). Our data indicate that infrequent users have a stronger relationship with negative mood. Our data suggested that those that use cannabis casually scored higher on the CES-D scale for depression, and consequently could be at greater risk for developing pre-depression symptomology compared to both chronic users and controls. Further implying that cannabis may not necessarily be an effective treatment for depressive symptoms but it may in fact contribute to deficits in emotion processing. However, it is important to note that cannabis use and its relationship to mood disorders is complex, Danielsson and colleagues (2016) showed that although cannabis use was comorbid with depression, other factors such as other abused substances and situational variables might have driven these effects. Unlike previous research on the effects of cannabis on anxiety we found that no significant effects on anxiety measures or pre-anxiety symptoms. This is contrary to previous research, for example Lai and colleagues (2015) showed a significant relationship between substance use disorder and anxiety as well as depression.

Previous research has supported the use of cannabis for a possible treatment for depression (Bambico et al., 2009; Micale et al., 2013; Zanettini et al., 2011) although this data is based on a particular phytocannabinoid, CBD which although present in recreational and medical cannabis and cannabis products sold in Colorado is so at varying degrees. It seems that the effects of cannabis on mood are still not clear. Confounds include the large range of phytocannabinoids users were exposed to, lack of control and understanding of these compounds, and their potential effects. This is further exacerbated by no current states offering cannabis as a medical solution for mood or anxiety disorders, despite Canada and the Netherlands supporting these conditions through their programs. A large international survey had 5.2% of respondents reporting cannabis use was a means to alleviate depressive symptoms (Hazekamp & Heerdink, 2013); a survey of medical users in California had 26.1% of participants self-reporting therapeutic benefits for depression and 37.8% for anxiety, despite California not recognizing either condition (Reinarman, Nunberg & Lanthier, 2011). This trend of self-medication for conditions other the one prescribed is too large to ignore when investigating the associations between cannabis use and mood disorders, increasing the need to include recreational users for research, especially when the casual user group are most likely recreational users and seem to sustain the greatest deficits in mood.

The discrepancies in both culture and research on the effects of cannabis on mood disorders do not go unnoticed by prescribing Colorado physicians: 64% suggested that cannabis use poses a threat to mental health and only 15% suggested that it might have mental health benefits. A vast majority of those surveyed (92%) agreed that continued education and research is needed (Kondrad & Reid, 2013). These discrepancies in data indicate that until there is more consistency in agreed treatments and regulations for cannabis use, the associations between cannabis use and mood disorders warrants further research. This study suggested that in an ecologically valid approach, where cannabis use was assessed as it is being used in the community, we saw a significant relationship between cannabis use and symptoms of mood disorders. Importantly, how cannabis use in the context of the informal unregulated model provides much needed insight into its effects on emotion processing.

We acknowledge our study has many limitations; for example, sample size (and resulting power), control for phytocannabinoids in terms of strength and type, confounding variables such as poly drug use, alcohol consumption, reliance on self-report, as a well as limited interpretation of depression due to a lack of clinical evaluation. Therefore, we are unable to determine a causal relationship between cannabis use and mood. Some of the differences we see between chronic and causal cannabis use could be a result of differences in group size. Despite the limitations, we feel that our data provides a starting point from which to design controlled experiments to further investigate the relationship between mood and cannabis use in a unique population. Whilst it lacks the power of the large cohort studies and longitudinal studies, the population we have access to has a unique perspective and experience with cannabis in a state legal model where cannabis use rates and consumption is very different from other state and countries.

Further research is needed to solidify associations between cannabis and mood/anxiety disorder symptoms and diagnoses.

## Conclusions

The relationship between cannabis exposure and mood is complex and our data suggests that in a post legalization model there is potential that less frequent exposure may contribute to negative mood states, in particular depressive symptoms. Further research is needed to better understand how state legal cannabis use effects mood especially in light of its relationship to depression.

##  Supplemental Information

10.7717/peerj.2782/supp-1 Data S1 Raw data for MANOVA and correlation analysis

10.7717/peerj.2782/supp-2 Supplemental Information 1 The recreational cannabis use evaluation

## Additional Information and Declarations

### Data Availability

The following information was supplied regarding data availability: The raw data has been supplied as a Supplemental File.
