# Supplementary material for: The relationship between cannabis use and measures of anxiety and depression in a sample of college campus cannabis users and non-users post state legalization in Colorado
Source: PeerJ. 2016 Dec 8;4:e2782. doi: 10.7717/peerj.2782 (PMC5149055; doi:10.7717/peerj.2782)
Supplement: Supplemental Information 1 [file peerj-04-2782-s002.pdf]

Recreational Cannabis Use Questionnaire. (R-CUE) Troup, Andrzejewski & Bastidas 2015

Complete the following and check the most appropriate response. Please answer each question honestly and to the best of your ability. You may add any extra information next to the question or on the back of each page.

1. **Age___**
2. **Are you part of Colorado's Medical Marijuana Registry (do you own a red card)?** ☐ Yes ☐ No
3. If you answered yes to #2, **for how long have you been a member of the registry?**

☐ N/A (Never a member)	☐ 2-4 years
☐ Less than one year	☐ 4-7 years
(This is my first red card)	☐ 7-10 years
☐ 1-2 years	☐ 10+ years

4. **How long ago did you first try cannabis?**

☐ N/A (Never tried)	☐ 2-4 years
☐ Less than one year	☐ 4-7 years
☐ 1-2 years	☐ 7-10 years
	☐ 10+ years

5. **How often do you use Cannabis (in any form)?**

☐ N/A (Don't use)	☐ A few (3-6) times a week
☐ Less than monthly (1-11 times/year)	☐ Daily
☐ Less than weekly (1-3 times/month)	☐ 2-4 times a day
☐ Once a week	☐ More than 4 times a day
☐ A couple (1-2) times a week	

6. **Have you ever used Cannabis more heavily than you do now?**
   - ☐ No, have never used.
   - ☐ No, same amount as now.
   - ☐ Yes. *How often did you use at that time?* ______________________________________

7. **Which of the following types of Cannabis and methods of intake do you prefer?** Check all bolded items that apply (*and check subcategories to the best of your knowledge/ability*)**.**

a. ☐ **N/A (Don't use)**

b. ☐ **Smoking Cannabis flower** (Bud, Nugget, etc.)

i. *Strain (type) of cannabis:*

☐ Indicas ("Body high")	
☐ Sativas ("Mind high")	
☐ Hybrids:	☐ Sativa-dominant
	☐ Indica-dominant
	☐ True hybrid (50/50 Sativa/Indica)

c. ☐ **Smoking Cannabis Concentrates** (Hashish/"Dabs")

i. *Strain (type) of cannabis:*

☐ Indicas ("Body high")	
☐ Sativas ("Mind high")	

☐ Hybrids:	☐ Sativa-dominant	
	☐ Indica-dominant	
	☐ True hybrid (50/50 Sativa/Indica)	

ii. ☐ Strain-specific hash. If so, list strains that you have used: ____

____ ____

____ ____

iii. *Method of THC extraction (type of concentrate):*

1. ☐ *Solvent based extraction:*
   - ☐ Butane Honey Oil (BHO)
   - ☐ Carbon Dioxide (CO_2)
   - ☐ Quick Wash Isopropyl Alcohol (QWISO)
   - ☐ Hexane solvent concentrates
   - ☐ Propane solvent concentrates
   - ☐ Ethanol solvent concentrates
   - ☐ "Shatter" hash (High purity butane/ethanol extraction)
2. ☐ *Solvent-less concentrates:*
   - ☐ Cold Water Extraction (CWE)/Icewax/Solvent-less wax/"grease"/"jewce"
   - ☐ Bubble hash
   - ☐ Screen filtered hash (Finger hash/Keif)

d. ☐ **Cannabis Edibles**

i.Specify:

☐ Pre-packaged baked edibles
☐ Hard candy/gummy edibles
☐ Chocolate edibles
☐ Drink-based edibles (THC-infused sodas, teas, etc.)
☐ Tinctures: ☐ Glycerin based
☐ Ethanol based
☐ Cannabis butter (Cannabutter)
☐ Other. Please list: ____

ii. *Cannabinoids contained:*

☐ CBD ☐ THC ☐ CBD/THC

e. ☐ **Dermal Cannabis Application:** ☐ Skin patches
☐ Lotions/balms/oils

8. In order of preference (1 being most preferred, 4 being least preferred), what is your preferred form of consuming Cannabis?

▪ Cannabis flower/nugget ▪ Concentrates/hash
▪ Edibles ▪ Topical/Dermal Absorption

☐ N/A (Don't use)

1. ____________________________________ (Most Preferred)
2. ____________________________________ (Second Most)
3. ____________________________________ (Second Least)

4. ______________________________________ (Least Preferred)

9. If applicable, **what smoking devices do you use? (Select all that apply)**

a. ☐ **None**

b. ☐ **Water-filtration devices:**

☐ Bong (upright/waterpipe)
☐ Bong (gravity)
☐ Bubbler

c. ☐ **Dry smoking devices:**

☐ Pipe (glass/metal)
☐ Steamroller
☐ Joint
☐ Blunt

d. ☐ **Vaporizers:**

☐ Bag vaporizers
☐ Whip vaporizers
☐ Portable/Pen vaporizers

e. ☐ **Dabs:**

☐ Spoon dabs
☐ Nail dabs
☐ Noodle dabs
☐ Health stone dabs
☐ Skillet dabs

10. If applicable, **in an average month, how much in Cannabis flower/nugget do you smoke?**

☐ **None** (0 grams)	☐ **An ounce or less** (14.1-28 grams)
☐ **A gram or less** (0-1 grams)	☐ **More than an ounce** (1-2 ounces)
☐ **An eighth of an ounce or less** (1.1-3.5 grams)	☐ **More than two ounces** (2-4 ounces)
☐ **A half of an ounce or less** (3.6-14 grams)	☐ **More than a quarter pound** (4+ ounces)

11. If applicable, **in an average month, how much in Cannabis concentrates do you smoke?**

☐ **None** (0 grams)	☐ **An ounce or less** (14.1-28 grams)
☐ **A gram or less** (0-1 grams)	☐ **More than an ounce** (1-2 ounces)
☐ **An eighth of an ounce or less** (1.1-3.5 grams)	☐ **More than two ounces** (2-4 ounces)
☐ **A half of an ounce or less** (3.6-14 grams)	☐ **More than a quarter pound** (4+ ounces)

12. If applicable, **in an average month, how many Cannabis edibles do you consume? (One edible is equal to what is considered one dose by the manufacturer).**

☐ **None**	☐ **4-8 edibles**
☐ **One edible**	☐ **8-20 edibles**
☐ **2-4 edibles**	☐ **20+ edibles**
